# Alcohol Consumption After Listing for Liver Transplantation Is Associated With Increased Risk of Alcohol Consumption After Transplantation

**DOI:** 10.1155/ijh/3221011

**Published:** 2025-06-26

**Authors:** Léa Abrial, Domitille Erard, Laure Tron, Sylvie Radenne, Agnès Bonadona, Justine Barthelon, Térésa Antonini, Marie Noelle Hilleret, Thomas Decaens, Jérôme Dumortier, Charlotte Costentin

**Affiliations:** ^1^Univ. Grenoble Alpes, Department of Hepato-Gastroenterology and Digestive Oncology, CHU Grenoble Alpes, Institute for Advanced Biosciences, CNRS UMR 5309-INSERM U1209, Grenoble, France; ^2^Departments of Hepatology, Hospices Civils de Lyon, University of Lyon, Lyon, France

**Keywords:** alcohol liver disease, alcohol relapse, liver transplantation

## Abstract

**Background:** Alcohol abstinence is required before liver transplantation (LT) for alcohol-related liver disease (ALD). However, some patients may have alcohol intake during the pretransplant period.

**Objectives:** Describe the prevalence of alcohol consumption on list and impact on the LT project.

**Methods:** All patients listed for ALD, in two French transplant centers, between January 2014 and December 2018 were included retrospectively. Documented alcohol consumption (DAC) on list was defined by any alcohol intake during the waiting period elicited by patient interview and/or by biology.

**Results:** Four hundred and twenty-six patients were included. DAC on list was observed in 41 patients (9.6%), with a median delay of 6.2 months (IQR 2.8; 11.4) after listing. Addiction counseling was proposed to 30 patients (73%), and 28 (68%) were placed or maintained in temporary contraindication. DAC on list was associated with the waiting time length (OR 1.07, 95% CI 1.04; 1.1; *p* < 0.001), occupation (“intermediate occupation” OR 6.39, 95% CI 1.93; 22.74, *p* = 0.003 and “employee”: OR 5.83, 95% CI 1.79; 20.68, *p* = 0.004 compared to “Craftsman” category) and less likely in former smokers (OR 0.23, 95% CI 0.07; 0.77; *p* = 0.02). We observed a higher risk of alcohol consumption after LT (OR 6.36, 95% CI 1.61–26.93; *p* = 0.009) in patient with DAC on list, but no impact on 5-year posttransplant survival.

**Conclusion:** Alcohol consumption on the list was documented in 9.6% of the patients, associated with an increased risk of alcohol consumption after LT. These results support systematic screening of alcohol consumption and active addiction counseling before transplant. Importantly, 5-year overall survival since listing was not statistically different between patients with or without DAC during the waiting time period.

**Summary**

• From 426 patients with alcohol-related liver disease listed for liver transplant, alcohol consumption was documented in 10% of patients awaiting liver transplantation (LT).
• Among them, a quarter was uncovered by a biological testing the day of liver transplant.
• Alcohol consumption on waiting time period was associated with alcohol consumption posttransplant.

## 1. Introduction

Alcohol liver disease (ALD) is an indication for LT since 1983. Ever since, ALD has become the leading indication for LT in Europe [1]. Alcohol abstinence is required before LT for alcohol-related liver disease. However, the risk of alcohol consumption recurrence after LT still raises concerns in the context of transplant shortage, with one donor for every 2.4 potential recipients in France [2]. While 5-year survival of patients transplanted for ALD is equivalent to that observed for other causes [3], recent studies suggest excessive posttransplant alcohol consumption is associated with reduced survival at 10 years [4, 5]. The definition of alcohol consumption relapse after a period of abstinence is not standardized, resulting into variability in reported rates of alcohol consumption after LT and impact on survival [6]. However, a consensus has emerged to distinguish between a major, sustained relapse (more than four drinks a day) and “slip” relapses defined by a single occasional drink followed by immediate return to abstinence [7, 8]. This distinction is critical and may partly explain the contradictory results regarding the impact of the alcohol consumption after LT on patient survival. If major and sustained alcohol consumption seems to be associated with a negative impact on survival [9], impact of “slips” has been poorly studied and may have little consequences [4, 7]. While many studies explored predictive factors of alcohol consumption relapse after transplantation and its impact on survival, data related to alcohol consumption during the waiting time period are scarce. Alcohol consumption while on the waiting list might concern 25% of patients listed for ALD [10]. There are no standardized procedures to screen and manage alcohol consumption during the waiting time period. In addition, there are no specific guidelines concerning the management of the transplant project. Some patients are discarded for transplantation or placed on temporary contraindication (TCI), based on the assumption that patients experiencing alcohol consumption during the waiting period have higher risk of posttransplant alcohol relapse. But there is little data available to support this hypothesis.

Therefore, the aim of this study was to describe the prevalence of alcohol consumption during the waiting time period and the impact on both the transplantation project and survival.

## 2. Participants and Methods

This was a French retrospective cohort study. All patients listed for liver transplant for ALD with the national transplant agency (Agence de Biomedicine (ABM)), in two transplant centers (Grenoble and Lyon), between January 2014 and December 2018, were included in the study. Patients transplanted during the same hospital stay of ALD diagnosis and without an alcohol abstinence period as an out-patient were excluded.

### 2.1. Study Design

Data were collected retrospectively from the medical records in each center and from the national transplant database (CRISTAL ABM). Data collection stopped on March 30, 2023. The study complied with ethical standards and the Helsinki Declaration of 1975, as revised in 2008. The study meets the ethics requirements of the CNIL (Commission Nationale de l'Informatique et des Libertés) Reference Methodology 004 related to research on data not involving human subjects (regulation reference CNIL1818709X) [11] not requiring ethics committee consultation according to French Law. Accordingly, the conduct of the study has been registered and authorized by Grenoble Alpes University Hospital Research Regulatory Board on November 21, 2022. Information was available to all patients, and objections to use personal data were recorded.

### 2.2. Definitions and Objectives

Diagnosis of ALD was considered when alcohol was recorded as one of the etiologies the ABM's database. Patients whose liver disease etiology discorded between medical records and ABM were excluded. The indication for LT as registered in the ABM by the referent physician was collected.

The primary objective was to describe the prevalence of alcohol consumption during the waiting time period. The primary endpoint was the rate of documented alcohol consumption (DAC) during the waiting time period. DAC on the list was defined as any alcohol consumption elicited during the waiting time period, documented either by medical interview and/or by biological screening with a positive blood or urine alcohol testing (> 0.10 g in the blood), including the day of LT.

Secondary objectives were (1) to describe the impact of DAC during the waiting time period on the transplant project; to this end, dates of TCI related to alcohol consumption on the list, addiction counseling and/or treatment, and/or removal from the waiting list were collected; (2) to assess determinants of DAC during the waiting time period based on demographic and clinical characteristics at listing; (3) to assess posttransplant alcohol consumption rates (posttransplant alcohol consumption was defined by any alcohol consumption elicited by medical interview, and/or by biological screening with a positive blood or urine alcohol testing); and (4) to describe overall survival according to alcohol consumption during the waiting time period and access to LT.

During the study period, Lyon Center relied on patient interview combined with monthly blood and urine alcohol testing to screen for alcohol consumption during the waiting time period. In Grenoble center, biochemical testing was performed upon suspicion of alcohol consumption on clinical interview. As alcohol quantitative assessment of alcohol intake could not be systematically recorded, patterns of alcohol consumption (casual or daily) during the waiting time period or posttransplant were determined according to the liver specialist qualifications in the medical records (or the addiction specialist if available).

### 2.3. Statistical Analysis

Demographic and clinical variables were described using headcounts and percentages for categorical variables, using mean (and standard deviation) and median for continuous variables.

Comparisons between patients with or without DAC during the waiting time period were made using the *χ*^2^ test (if sufficient number of cases were available) or the Fisher test for categorical variables and using Student's *t* test (if the assumptions of normality and equality of variances were met) or the Wilcoxon test for continuous variables.

Logistic regression models were used to determine baseline variables associated with DAC during the waiting time period, including center, sex, age, socioprofessional category, family status, tobacco consumption, addiction follow-up, length of abstinence at listing, presence of psychiatric disorders, metabolic risk factors or liver comorbidities, MELD score, indication for transplantation (decompensated liver disease or hepatocellular carcinoma), and time on the waiting list. When investigating factors associated with posttransplant alcohol consumption, the variable “alcohol consumption during waiting period” was also considered.

Factors associated with *p* value ≤ 0.2 in univariate analysis were included in the final multivariate model. An association with a *p* value of less than 0.05 was considered statistically significant in the multivariate analyses. A sensitivity analysis was performed, restricting the study population to patients with a biochemical alcohol test during the waiting time period.

Overall survival (to death) was assessed using the Kaplan–Meier estimator. Time zero was the date of listing, censoring occurred at loss to follow-up, or March 30, 2023, whichever came first. Survival was compared between patients with and patients without DAC during the waiting period, using the log-rank test. Factors associated with survival were studied using the Cox model. However, proportional hazard hypothesis was not verified for all the covariates. Therefore, we adjusted the Cox model with time-varying effects and other constant effects. Potential predictive factors of survival were studied in a univariate model (center, sex, age, socioprofessional category, family situation, tobacco consumption, addiction follow-up, duration of abstinence, psychiatric disorders, metabolic risk factors, liver comorbidities, MELD score, transplantation indication, duration on waiting list, transplantation, and alcohol relapse during waiting period). Final multivariate survival model included center, sex, and age as well as variables with a *p* value < 0.2 in the univariate analyses and which remained associated with survival with a *p* value < 0.2 in the multivariate model (this was done in order to limit number of variables in the final multivariate model and the risk of collinearity).” Pretransplant addiction counseling variable was not retained despite *p* < 0.2 to avoid collinearity with “center” variable.

For transplanted patients, posttransplant survival was studied using the Kaplan–Meier estimator and the log-rank test, comparing patients with or without documented relapse of alcohol consumption during the waiting time period and posttransplant. Factors associated with posttransplant survival were investigated using Cox proportional effects model (hypothesis validated for all covariables). Final multivariate model included center, gender, age, and variables with a *p* value < 0.2 in univariate analysis. The absence of collinearity was checked in all the multivariate models. Analyses were performed using R software (4.2.2).

## 3. Results

**Figure 1:**
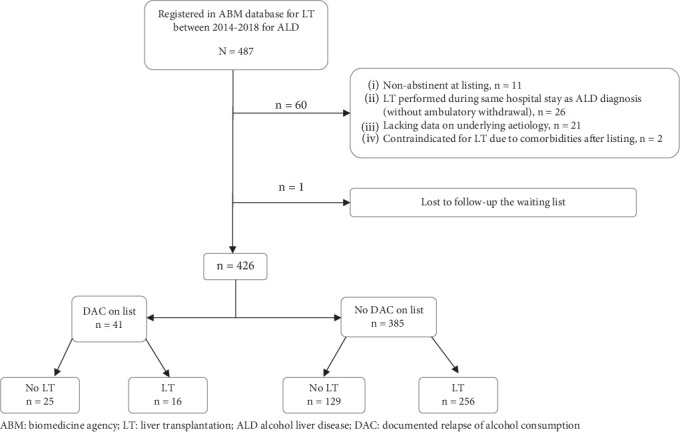
Flowchart. ABM: biomedicine agency; LT: liver transplantation; ALD: alcohol liver disease; DAC: documented relapse of alcohol consumption.

Four hundred and eighty-seven patients with ALD were registered within the ABM database during the study period. Sixty-one patients were excluded (Figure 1). Analysis was performed on a study population of 426 patients. Patient's characteristics are described in Table 1. DAC during the waiting time period was observed in 41 patients (9.6%), with a median delay of 6.2 months (IQR 2.8; 11.4) after listing (Table 2). Alcohol consumption was considered casual in 19 patients and daily in 14, based on the qualification of the referring liver or addiction specialist (data missing in 8 patients). Patients with DAC during the waiting time period, compared to patients without, were less often from the occupational category “craftsman, shopkeeper, and company director” and more often former smokers (respectively, 27.8% vs. 54%; *p* < 0.011 and 74.4% vs. 84.1%; *p* < 0.002).

Patterns and management of DAC are detailed in Table 2. Alcohol consumption was detected by interview in 18 patients (43.9%) and through biological tests in 21 patients (51.2%). In the remaining two patients, alcohol consumption was revealed by an alcohol withdrawal syndrome in one and alcohol consumption reported by the family after liver biopsy suggesting acute alcohol consumption in the other. Among the 21 patients whose alcohol consumption was elicited by biology, the test was carried out the day of a call for transplantation in 10 patients (24.4%), canceling the procedure in seven cases. An addiction counseling was proposed to 30 patients (73%), and 23 (56.1%) had at least one after documentation of the alcohol consumption, 14 of whom had more than one counseling. Of the 18 patients who did not have an addiction counseling after documentation of the alcohol consumption, 8 of the 11 patients with available data (72.7%) had a daily consumption. After exclusion of 3 patients transplanted with positive testing, 6 patients with missing data, of the 32 remaining patients, 15 patients (46.9%) returned to sustained abstinence during follow-up. Twenty-eight (68.3%) from the 41 patients were placed or remained on TCI for alcohol consumption. TCI was lifted after a mean of 5.1 (IQR 2.2; 7.3) months of abstinence. Of the 13 patients (31.7%) who were not placed on TCI, 6 were classified as casual drinkers and 3 as daily drinkers by the referring hepatologist (missing data for four). Patients with alcohol consumption during the waiting time period were less likely to be transplanted than patients without (39.0% and 66.5%, respectively (*p* < 0.001)). Among the 41 patients with alcohol consumption during the waiting time period, 16 (39.0%) were transplanted (3 despite positive test the day of the call and 12 who had returned to abstinence et one nonabstinent), 6 patients (14.6%) were ruled out of waiting list due to alcohol consumption, 14 (34.2%) were removed for worsening or death, and 2 (4.9%) were removed for improvement. Three patients were still waiting for transplant at the time of analysis. Trajectories of patients with DAC during the waiting period are summarized in Figures S1 and S2.

### 3.1. Determinants of DAC During the Waiting Time Period

We investigated features associated with DAC during the waiting time period (Table 3). The length of the waiting period was significantly associated with DAC, with an increasing odd ratio for waiting periods from 6 to 12 months and more than 12 months (OR 4.75, 95% CI [1.74–15.17] and 7.93, 95% CI [3.16–24.17], respectively) (Table S1). In multivariate analysis, the variables associated with DAC were: length of time on the waiting list (OR 1.07, 95% CI [1.04–1.1], *p* < 0.001), socioprofessional category, “intermediate occupation” and “employee” compared to the “craftsman, shopkeeper, and company director” (OR 6.39 95% CI 1.93; 22.74; *p* = 0.003 and 5.83 95% CI 1.79; 20.68; *p* = 0.004, respectively) and being former smoker versus never smoker (OR 0.23, 95% CI [0.07; 0.77], *p* = 0.017).

### 3.2. Alcohol Consumption After LT

A total of 272 patients were transplanted. Alcohol consumption after transplantation was documented in 49 patients, after a median time of 24.9 months (IQR 16.3; 40.4). It was qualified as casual in 23 patients and daily in 25 (1 missing data). Analysis of factors associated with alcohol consumption after LT is presented in Table 4. DAC during the waiting time period was significantly associated with alcohol consumption after LT in multivariate analysis (OR of 6.36 95% CI [1.61; 26.93], *p* < 0.009), independent of consumption pattern (casual or daily). Other factors associated with alcohol consumption after LT were as follows: female sex (OR 0.26, 95% CI [0.06; 0.86]), abstinence length at listing (OR 0.95 95% CI [0.91; 0.98]), and presence of psychological disorders (OR 0.24 95% CI [0.06; 0.81]). Of the 3 patients transplanted despite a positive blood alcohol level the day of transplant, 1 died before discharge, and the other 2 remained abstinent after LT.

A sensitivity analysis was performed on the restricted population of patients who had undergone at least one biological testing during the waiting time period, including the day of LT (Table S2). All associations were confirmed.

### 3.3. Survival Analysis

**Figure 2:**
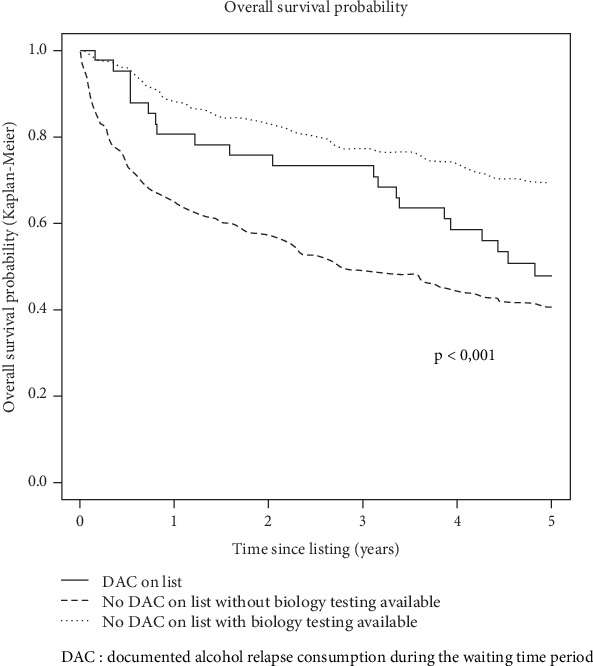
Probability of survival since listing stratified according to alcohol consumption and biological testing status during the waiting time period.

When considering the whole study population (*n* = 426), 5-year overall survival since listing was not statistically different between patients with DAC during the waiting time period (*n* = 41: 47.8% 95% CI [34.5; 66.2]) and patients without (*n* = 385: 55.1% 95% CI [50.2; 60.4], *p* = 0.710). To further explore this result, the study population was divided into three groups: (1) DAC during the waiting time period, (2) no DAC in patients with biology testing available, and (3) no DAC in patients without biology testing available (*n* = 184). The last group had the worst 5-year survival rates (Group 1 (*n* = 41): 47.8%, 95% CI [34.5; 66.2], Group 2 (*n* = 201): 68.7%, 95% CI [62.4; 75.7], and Group 3 (*n* = 184): 40.3%, 95% CI [33.7; 48.2]) *p* < 0.001) (Figure 2).

**Figure 3:**
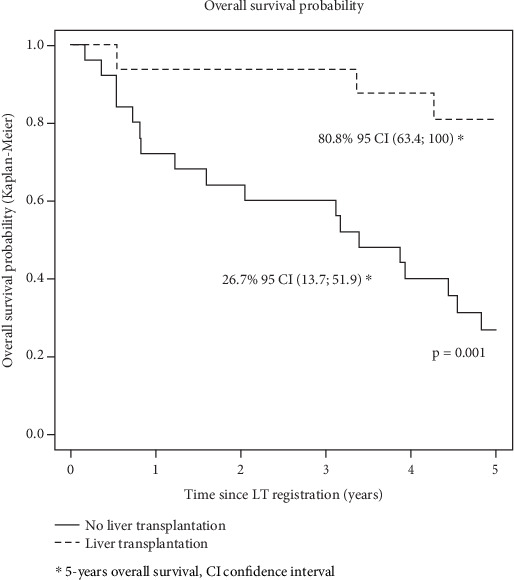
Probability of survival since listing for patient with documented alcohol consumption during the waiting time period stratified according to access to liver transplantation.

In an analysis limited to patients with DAC during the waiting time period (*n* = 41), 5-year survival since listing was 80.8% (95% CI [63.4; 100.0]) for transplanted patients compared to 26.7% (95% CI [13.7; 51.9]) for nontransplanted patients (*p* = 0.001) (Figure 3).

**Figure 4:**
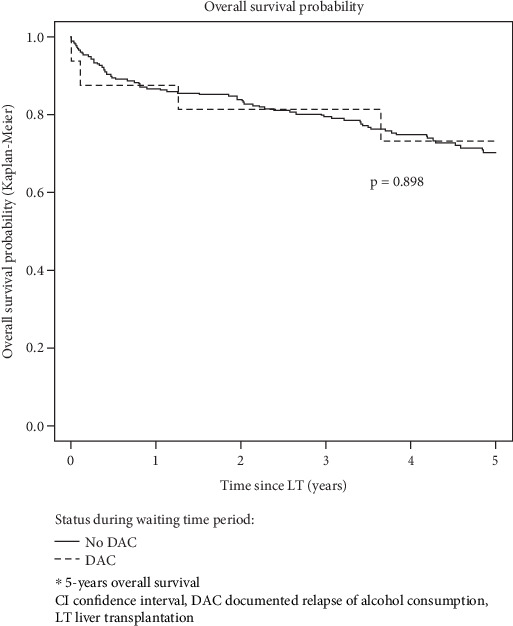
Probability of survival after LT stratified according to documented alcohol consumption status during the waiting time period.

Finally, when restricting the population to the 272 patients subsequently transplanted, 5-year overall survival after transplant was not statistically different between patients with DAC during the waiting time period (*n* = 16: 73.1, 95% CI [53.5; 100]) and patients without (*n* = 256: 70.1, 95% CI [64.3; 76.4]) *p* = 0.898) (Figure 4).

## 4. Discussion

From our cohort of 426 patients with ALD listed for liver transplant in two French centers, alcohol consumption during the waiting time period was documented in 9.6% of the patients. Alcohol consumption during the waiting time period was more likely in the case of longer waiting time periods and occupation “intermediate employee” and less likely in former smokers. DAC on the list increased the risk of alcohol consumption after LT but had no impact on 5-year overall survival after LT.

In the literature, prevalence of alcohol consumption during the waiting time period is reported to be as high as 25% of patients, more than twice the prevalence observed in our study [10]. This discrepancy could be related to the modalities of screening for alcohol consumption. In our study, assessment of alcohol consumption was mostly reliant on the liver specialist. However, it has been shown that hepatologist was less performant compared to addiction specialists to detect alcohol consumption relapse [12]. Reassessment of alcohol consumption was not systematically recorded in medical reports from referring hepatologists. When alcohol consumption was recorded, the assessment methods were not standardized or clearly codified, particularly the information on patterns of consumption (casual vs. daily) or quantitative assessment (in grams per day or per week) were frequently missing, making it difficult to analyze alcohol consumption trajectories and to qualify the severity of alcohol consumption. Importantly, half of DAC on list were detected only by random biological screening. Moreover markers used in this study were less sensitive than those currently available, such as plasma phosphatidylethanol or urinary ethylglucuronide [13]. Importantly, in 2016, the International Liver Transplant Society (ILTS) issued recommendations regarding ALD patients and LT, encouraging monitoring for alcohol use by clinical interviewing and random biochemical testing. Interestingly, in a recent study reporting the use of ethyl glucuronide to detect harmful alcohol consumption in patients with presumed metabolic fatty liver disease, positive results in patients who alleged no alcohol consumption consistently led the patient to acknowledge alcohol consumption and initiate a discussion with the medical team [14]. Therefore, more systematic random testing using specific tests could be an opportunity to uncover alcohol consumption in patients listed for LT, initiate discussion on alcohol and implement addiction management.

In our study, there were few actionable factors associated with DAC during the waiting time period. Longer duration on the waiting list was associated with alcohol consumption, further suggesting screening for alcohol relapse is critical all along the patient pathway. ILTS guidelines also emphasized that patients with ALD considered eligible for LT needed to be assessed and managed by an alcohol specialist, and psychosocial interventions should be routinely used in the medical management of these patients in order to properly recognize patients most at risk of alcohol consumption and implement adequate addiction support susceptible to increase chances to sustain abstinence [15, 16]. Donnadieu-Rigole et al. highlighted the added value of an addiction specialist to identify patients at risk of severe relapse and arrange for specific follow-up aiming at improving addiction and LT outcomes [12].

Unfortunately few data could be collected on patient's psychosocial and addiction profile: Age of first alcohol consumption, consumption duration, associated addictions except tobacco, and history of addiction treatment were not included in medical records in approximately 80% of cases. These data are, however, critical to understand patients history and uncover limiting factors to the definitive abstinence objective required in a transplant project [12, 17]). This may suggest that hepatologists are not sufficiently aware of addiction issues and ways to interview patients regarding these matters. Also, patient's perspective of the transplant project could not be assessed: fears with respect to the risk of the procedures, anxiety related to a foreign organ or even guilt toward the donor. All these feelings could be potential contributors to the risk of alcohol consumption.

Importantly, 51.2% of DAC during the waiting time period was detected only by biological screening, including 24.4% on the day of a call for transplantation. In this study, out of the 170 tests performed the day of a call for LT, 10 were positive (6%). Three patients underwent transplantation while blood sample was positive for alcohol the day for transplantation calling. For two of them, there was no documentation in the medical records to characterize the team assessment of this result. Regarding the last patient, the team considered it was a slip and moved on to the procedure. It is noteworthy that none of these three patients consumed alcohol on posttransplantation period. Positive testing the day of a call for LT may trigger concerns about sustained abstinence following LT. However, positive testing the day of a call does not necessary mean sustained harmful alcohol consumption on list. Facing a traumatic event (call for transplant), some patients might be tempted to take “one last drink before transplant.” Importantly, there is no evidence of a reduction in long-term survival in case of alcohol consumption identified by systematic testing the day of LT [18].

Evidence of DAC during the waiting time period resulted in 68.3% of patients being placed on a TCI for LT for approximately 6.8 months. This suggests the majority of hepatologists believe that a further period of abstinence before LT is mandatory. However, TCI was not systematically implemented even in case of daily consumption. Referent hepatologists may also minimize the meaning of alcohol relapse on waiting period. However, in our study, DAC during the waiting time period, even followed by return to abstinence in most patients later transplanted, was nevertheless associated with alcohol consumption after liver transplant. Without impact on 5-year survival, alcohol consumption during the waiting time period should not lead to question the relevance of LT in these patients. However, our results stress out the need to strengthen addiction support to all patients with ALD awaiting LT, in order to uncover casual consumption and prevent relapse of sustained alcohol consumption, which is known to be associated with recurrence of alcohol-related liver disease in the graft, with negative impact on long-term survival [4].

There are limitations to this work, inherent to the retrospective design. Some data could not be retrieved, such as addiction history and amount of alcohol intake at the time of relapse or cause of death. Importantly, reassessment of alcohol consumption was not systematically collected or, if collected, not in a standardized manner, suggesting the urgent need for standardization of data recorded regarding alcohol intake in the transplant setting. It is also likely that some patients had alcohol consumption during the waiting period without being identified. It is interesting to see that among the group of patients considered “abstinent,” patients who had random and negative biological testing had better survival compared to patients without any biological testing over the waiting time period. Even more, these patients experienced worse survival compared to patients with documented relapse during the waiting time period. It is plausible some patients had ongoing alcohol consumption and missed the opportunity to be included in addiction programs associated with higher chances to reach abstinence and improve liver function [10, 19]. Also, data only relate the practices of two centers, and the small number of patients with DAC during the waiting time period limited statistical power on predictive factors and survival.

Our results, however, highlight the need to improve data collection on patient alcohol history, alcohol consumption patterns, and social support in order to identify the most vulnerable candidates and tailor the management of patients according to their needs in order to improve the long-term outcomes of LT in ALD patients [20]. This was also a strong recommendation from the ILTS working group [16].

In conclusion, DAC concerned almost 10% of patients awaiting LT for alcohol-related liver disease in our cohort. Nearly a quarter of the patients with DAC during the waiting time period were uncovered by a biological testing the day of a call for liver transplant. Alcohol consumption during the waiting time period was associated with alcohol consumption posttransplant, but no impact on 5-year posttransplant survival was observed. Consequently, improving screening for alcohol consumption is critical and should be associated with multidisciplinary management, including addiction specialists within the transplant team. Prospective studies are needed to investigate the impact of enhanced addiction support on the risk of alcohol consumption before and after LT and the impact on survival.

## Figures and Tables

**Table 1:** Patient's characteristics (*N* = 426).

	**Documented alcohol consumption on list**	**No documented alcohol consumption on list**	**p** ** value**
Center *n* (%)			
Grenoble	15 (36.6)	170 (44.2)	0.353
Lyon	26 (63.4)	215 (55.8)	
Sociodemographic variables			
Men %	34 (82.9)	337 (87.5)	0.554
Age, years, mean (SD)	55.8. (± 8.8)	58.2. (± 6.8)	0.139
Main occupational status *n* (%)			**0.011**
Craftsman, shopkeeper, company director	10 (27.8)	169 (54.0)	
Production and farmer	2 (5.6)	22 (7.0)	
Executive, higher intellectual profession	3 (8.3)	9 (2.9)	
Intermediate occupation	11 (30.6)	43 (13.7)	
Employee	9 (25.0)	59 (18.8)	
Worker	1 (2.8)	11 (3.5)	
Partner yes *n* (%)	22 (56.4)	245 (66.4)	0.212
Children yes *n* (%)	29 (78.4)	287 (77.4)	0.493
Addictions			
Smoking *n* (%)			**0.002**
Never	10 (25.6)	59 (15.9)	
Current	21 (53.9)	129 (34.8)	
Former	8 (20.5)	183 (49.3)	
Pretransplant addiction visit *n* (%)	20 (50.0)	159 (41.5)	0.301
Abstinence duration before listing, months, median (p25; p50)	8.8 (4.8; 13.7)	10.7 (5.8; 25.1)	0.056
Comorbidities			
Psychiatric disorder *n* (%)			0.363
Depressive disorder	8 (19.5)	54 (14.2)	
Psychotic disorder	5 (2.4)	5 (1.3)	
Hypertension n (%)	11 (26.8)	132 (34.3)	0.337
Diabetes *n* (%)	10 (24.4)	124 (32.2)	0.305
Body mass index, mean (SD)	26.0 (± 5.2)	27.0 (± 5.1)	0.095
Underlying liver diseases n (%)			
Only alcohol	25 (61.0)	257 (66.8)	0.321
Alcohol + dysmetabolic^a^	7 (17.1)	78 (20.3)	
Alcohol + viral	7 (17.1)	31 (8.1)	
Alcohol + others	1 (2.4)	6 (1.6)	
Several liver comorbidities	1 (2.4)	13 (3.4)	
Hepatocellular carcinoma *n* (%)	23 (56.1)	196 (50.9)	0.527
Liver disease severity			
MELD, median (SD)	14.7 (± 6.3)	15.0 (± 8.5)	0.217
Transplantation indication *n* (%)			
Decompensated cirrhosis	18 (43.9)	196 (50.9)	0.394
Hepatocellular carcinoma	23 (56.1)	189 (49.1)	
Time on waiting list, months, median (SD)	14.2 (7.5; 36.1)	6.2 (1.7; 12.1)	**< 0.001**
Biological test for AC *n* (%)			**< 0.001**
No	8 (19.5)	184 (47.8)	
One	6 (14.6)	87 (22.6)	
Several	27 (65.9)	114 (29.6)	
Listing status at endpoint *n* (%)			**< 0.001**
Delisted for patient's personal decision	0 (0.0)	2 (0.5)	
Delisted to documented alcohol relapse	6 (14.6)	0 (0.0)	
Delisted for worsening	5 (12.2)	37 (9.4)	
Delisted for improvement	2 (4.9)	14 (3.6)	
Temporary contra indication	3 (7.3)	10 (2.6)	
Death	9 (22.0)	67 (17.4)	
Liver transplantation	16 (39.0)	256 (66.5)	**< 0.001**
Post-LT follow-up, months, median (p25; p50)	52.8 (35.3; 69.6)	52.7 (33.2; 66.8)	0.758
Alcohol relapse post LT %	8 (57.1)	42 (17.8)	**0.002**

*Note:* Bold data indicates significant *p* value.

Abbreviations: IQR, interquartile range; LT, liver transplantation; MELD, model end-stage liver disease; SD, standard deviation.

^a^High blood pressure and/or diabetes and/or obesity.

**Table 2:** Patterns and management of documented alcohol consumption (*N* = 41).

**Pattern of alcohol consumption, median (IQR)**	
Time between listing and relapse, months	6.2 (2.8; 11.4)
Time between withdrawal and relapse, months, (7% MD)	19 (9.8; 37.0)
Modality of consumption, *n* (%) (on 33 patients)^a^	
Occasional	19 (57.6)
Daily	14 (42.4)
Documentation method, *n* (%)	
Interview +/− biological	18 (43.9)
Biological alone, except the day of a call for transplant	11 (26.8)
Biology the day of a call for transplant	10 (24.4)
Incidental	2 (4.9)
Addiction treatment, *n* (%)	
Proposition of addiction counseling	30 (73,2)
Effective addiction counseling	23 (56,1)
1	9 (22,0)
>1	14 (34.1)
Temporary contraindication (TCI) for DAC on list	
*n* (%)	28 (68.2)
Duration, months, mean (SD)	13.5 (13.2)
Return to abstinence (32 patients with data available)^b^	
No or 2^nd^ relapse during the waiting period, *n* (%)	17 (53.1)
Yes *n* (%)	15 (46.9)
Time between return to abstinence and TCI lift, months, median (IQR)	5.1 (2.2; 7.3)
Reason for delisting, *n* (%)	
Worsening	5 (12.2)
Improvement	2 (4.9)
Deaths	9 (22.0)
Liver transplantation	16 (39.0)
DAC on list	6 (14.6)
Still on waiting list for LT	3 (7.3)

Abbreviations: DAC: documented alcohol consumption; IQR: interquartile range; LT: liver transplant; MD: missing data; SD: standard deviation; TCI: temporary contraindication.

^a^After exclusion of eight patients with missing data.

^b^After exclusion of three patients transplanted with positive testing and six patients with missing data.

**Table 3:** Factors associated with documented alcohol consumption during the waiting time period list (*N* = 426).

	**Univariate**				**Multivariate**		
	**OR**	**95% CI**	**p** ** value**	**Global ** **p** ** value**	**ORa**	**95% CI**	**p** ** value**
Center (ref: Grenoble)	1.37	[0.71; 2.73]	0.354		1.82	[0.74; 4.76]	0.205
Gender (ref: Men)	1.45	[0.56; 3.27]	0.405		0.9	[0.27; 2.66]	0.858
Age (years)	0.96	[0.92; 1]	**0.04**		0.99	[0.93; 1.06]	0.763
Main occupational status (ref: Craftsmen^a^)				0.018			
Production and farmer	1.54	[0.23; 6.33]	0.595		1.77	[0.13; 12.12]	0.605
Executive, higher intellectual profession	5.63	[1.12; 22.61]	**0.02**		6.18	[0.75; 36.7]	0.055
Intermediate occupation	4.32	[1.72; 11.03]	**0.002**		6.39	[1.93; 22.74]	**0.003**
Employee/worker	2.41	[0.95; 6.13]	0.06		5.83	[1.79; 20.68]	**0.004**
Partner (ref: Yes)	0.65	[0.34; 1.29]	0.215				
Children (ref: Yes)	1.06	[0.49; 2.56]	0.89				
Smoking (ref: Never)				0.002			
Current	0.96	[0.43; 2.25]	0.923		0.9	[0.32; 2.64]	0.839
Former	0.26	[0.09; 0.68]	**0.006**		0.23	[0.07; 0.77]	**0.017**
Addiction counseling in pre-LT assessment (ref: Yes)	1.41	1.41	[0.73; 2.72]	0.303			
Length of abstinence at listing (months)	0.98	[0.96; 1]	0.081		0.99	[0.96; 1]	0.193
Past or present mental disorder (ref: Never)				0.57			
Depressive disorder	1.49	[0.61; 3.27]	0.344				
Psychotic disorder	2.01	[0.1; 12.99]	0.529				
Current high blood pressure (ref: Yes)	0.7	[0.33; 1.41]	0.338				
Current Types 1 or 2 diabetes (ref: Yes)	0.68	[0.31; 1.38]	0.308				
Body mass index	0.96	[0.89; 1.02]	0.203				
Liver comorbidity (ref: No)				0.495			
Metabolic^b^	0.92	[0.36; 2.11]	0.857				
Viral	2.32	[0.87; 5.58]	0.072				
Other	1.71	[0.09; 10.59]	0.625				
Several	0.79	[0.04; 4.23]	0.825				
Past or present hepatocellular cancer (ref: Yes)	1.23	[0.65; 2.38]	0.528				
MELD score	0.96	[0.92; 1.01]	0.11		0.99	[0.93; 1.04]	0.639
LT indication (ref: cirrhosis decompensation)	1.33	[0.69; 2.56]	0.395				
Time on waiting period (in months)	1.06	[1.04; 1.08]	**< 0.001**		1.07	[1.04; 1.1]	**< 0.001**

*Note:* Bold data indicates significant *p* value.

Abbreviations: 95% CI: 95% confidence interval; LT: liver transplantation; MELD: model end-stage liver disease; OR: odds ratio; ORa: adjusted odds ratio; Ref: reference.

^a^Craftsmen, shopkeeper, and company director.

^b^High blood pressure and/or diabetes and/or obesity.

**Table 4:** Factors associated with posttransplant alcohol consumption (*N* = 244).

	**Univariate**				**Multivariate**		
	**OR**	**95% CI**	**p** ** value**	**Global ** **p** ** value**	**ORa**	**95% CI**	**p** ** value**
Center (ref: Grenoble)	0.78	[0.41; 1.49]	0.445		1.74	[0.57; 5.2]	0.324
Gender (ref: Men)	0.37	[0.09; 1.11]	0.117		0.26	[0.06; 0.86]	**0.047**
Age (years)	0.95	[0.9; 0.99]	**0.012**		0.96	[0.91; 1.01]	0.09
Main occupational status (ref: Craftsmen^a^)				0.363			
Production and farmer	0.48	[0.07; 1.88]	0.352				
Executive, higher intellectual profession	0.48	[0.02; 2.89]	0.501				
Intermediate occupation	1.36	[0.57; 3.13]	0.47				
Employee/worker	0.7	[0.27; 1.67]	0.443				
Partner (ref: Yes)	0.85	[0.44; 1.67]	0.631				
Children (ref: Yes)	0.61	[0.3; 1.31]	0.193		0.56	[0.24; 1.32]	0.173
Smoking (ref: Never)							
Current	0.77	[0.33; 1.83]	0.549				
Former	0.64	[0.28; 1.47]	0.279				
Addiction counseling in pre-LT assessment (ref: Yes)	0.62	[0.32; 1.18]	0.152		0.35	[0.11; 1.09]	0.065
Length of abstinence at listing (in months)	0.96	[0.93; 0.98]	**0.007**		0.95	[0.91; 0.98]	**0.013**
Past or present mental disorder (ref: Never)	0.42	[0.12; 1.13]	0.119		0.24	[0.06; 0.81]	**0.035**
Current high blood pressure (ref: Yes)	0.62	[0.29; 1.26]	0.204				
Current Types 1 or 2 diabetes (ref: Yes)	0.48	[0.21; 0.99]	0.056		0.73	[0.3; 1.72]	0.485
Body mass index	0.97	[0.9; 1.03]	0.352				
Liver comorbidity (ref: No)				0.727			
Metabolic^b^	0.64	[0.26; 1.42]	0.297				
Viral	0.74	[0.2; 2.12]	0.605				
Other/Several	0.78	[0.12; 3.21]	0.759				
Past or present hepatocellular carcinoma (ref: Yes)	1.03	[0.55; 1.94]	0.924				
MELD score	1.03	[0.99; 1.07]	0.167		1.03	[0.98; 1.08]	0.249
Liver transplant indication (ref: Decompensated cirrhosis)	0.81	[0.42; 1.51]	0.503				
Hepatocellular carcinoma							
Time on waiting period (months)	0.99	[0.95; 1.02]	0.587				
Documented alcohol consumption on list	6.15	[2.03; 19.59]	**0.001**		6.36	[1.61; 26.93]	**0.009**

*Note:* Bold data indicates significant *p* value.

Abbreviations: 95% CI: 95% confidence interval; LT: liver transplantation; MELD: model end-stage liver disease; OR: odds ratio; ORa: adjusted odds ratio.

^a^Craftsmen, shopkeeper, and company director.

^b^High blood pressure and/or diabetes and/or obesity.

## Data Availability

The data that support the findings of this study are available from the corresponding author upon reasonable request.

## Nomenclature

ALD: alcohol liver disease
LT: liver transplantation

## Supporting Information

Supporting Information Additional supporting information can be found online in the Supporting Information section. Table S1. Association between waiting period length and documented alcohol consumption during the waiting time period (*N* = 426). Table S2. Factors associated with documented alcohol consumption during the waiting time period, excluding patients who did not have a bioassay (*N* = 242). Figure S1. Patients trajectories (patients with documented alcohol consumption on list excluding patients with alcohol consumption only documented by positive testing the day of a call for LT) (*N* = 31). Figure S2. Patients trajectories (patients with alcohol consumption only documented by positive testing the day of a call for LT) (*N* = 10).
