# Supplementary material for: Alcohol Consumption After Listing for Liver Transplantation Is Associated With Increased Risk of Alcohol Consumption After Transplantation
Source: Int J Hepatol. 2025 Jun 26;2025:3221011. doi: 10.1155/ijh/3221011 (PMC12226167; doi:10.1155/ijh/3221011)
Supplement: Supporting Information — Additional supporting information can be found online in the Supporting Information section. Table S1. Association between waiting period length and documented alcohol consumption during the waiting time period (N = 426). Table S2. Factors associated with documented alcohol consumption during the waiting time period, excluding patients who did not have a bioassay (N = 242). Figure S1. Patients trajectories (patients with documented alcohol consumption on list excluding patients with alcohol consumption only documented by positive testing the day of a call for LT) (N = 31). Figure S2. Patients trajectories (patients with alcohol consumption only documented by positive testing the day of a call for LT) (N = 10). [file 3221011.f1.docx]

**Supplementary materiel**

|  | **Alcohol relapse on waiting period** | | | | | | | |
| --- | --- | --- | --- | --- | --- | --- | --- | --- |
|  | **No** | **Yes** | |  | | Univariate analysis | | |
| **Waiting time duration** | *%* | | *%* | *p-value* | *OR* | | *95% CI* | *p-value* |
| <6 months | 47 | | 12.2 | <0,001 | 1 (reference) | |  |  |
| 6-12 months | 25.7 | | 31.7 |  | 4.75 | | [1.74-15.17] | 0.004 |
| ≥12 months | 27.3 | | 56.1 |  | 7.93 | | [3.16-24.17] | <0.001 |
| 95% CI: 95% confidence interval; OR: Odds Ratio | | | | | | | | |

**Supplementary Table 1. Association between waiting period length and documented alcohol consumption during the waiting time period (N=426)**

**Supplementary Table 2. Factors associated with documented alcohol consumption during the waiting time period, excluding patients who did not have a bioassay (N=242)**

|  |  | **Univariate** | | | |  | **Multivariate** | | |
| --- | --- | --- | --- | --- | --- | --- | --- | --- | --- |
|  |  | **OR** | **95% CI** | **p.value** | **global p.value** |  | **ORa** | **95% CI** | **p.value** |
| **Center** (ref : Grenoble) |  | 0.32 | [0.15;0.67] | **0.002** |  |  | 0.58 | [0.14;2.26] | 0.436 |
| **Gender** (ref : men) |  | 1.22 | [0.46;2.88] | 0.665 |  |  | 0.59 | [0.17;1.83] | 0.382 |
| **Age** (in years) |  | 0.98 | [0.93;1.02] | 0.309 |  |  | 1.02 | [0.95;1.09] | 0.635 |
| **Main occupational status** (ref : Craftsmen*) |  |  |  |  |  |  |  |  |  |
| Production and farmer |  | 1.12 | [0.16;4.81] | 0.891 |  |  | 1.86 | [0.15;13.62] | 0.574 |
| Executive, higher intellectual profession |  | 4.2 | [0.79;18.8] | 0.067 |  |  | 4.96 | [0.58;31.91] | 0.102 |
| Intermediate occupation |  | 3.42 | [1.31;9.1] | **0.012** |  |  | 6 | [1.71;22.85] | **0.006** |
| Employee/worker |  | 2.71 | [1.02;7.23] | **0.044** |  |  | 6.66 | [1.89;25.95] | **0.004** |
| **Partner** (ref : yes) |  | 0.73 | [0.36;1.47] | 0.369 |  |  |  |  |  |
| **Children** (ref : yes) |  | 1.02 | [0.45;2.54] | 0.961 |  |  |  |  |  |
| **Smoking** (ref : never) |  |  |  |  |  |  |  |  |  |
| Current |  | 0.77 | [0.33;1.9] | 0.563 |  |  | 0.87 | [0.3;2.69] | 0.799 |
| Former |  | 0.24 | [0.09;0.67] | **0.007** |  |  | 0.27 | [0.07;1.01] | 0.053 |
| **Addiction counselling in pre-LT assessment** (ref : yes) | 0.58 | | [0.29;1.15] | 0.118 |  |  | 0.86 | [0.26;3.06] | 0.803 |
| **Length of abstinence at listing (in months)** |  | 0.98 | [0.95;1] | 0.054 |  |  | 0.98 | [0.96;1] | 0.164 |
| **Past or present mental disorder** (ref : never) |  | 1.29 | [0.51;2.94] | 0.837 |  |  |  |  |  |
| **Current high blood pressure** (ref : yes) |  | 0.75 | [0.34;1.55] | 0.453 |  |  |  |  |  |
| **Current type 1 or 2 diabetes** (ref : yes) |  | 0.62 | [0.27;1.29] | 0.219 |  |  |  |  |  |
| **Body mass index** |  | 0.95 | [0.87;1.02] | 0.143 |  |  | 0.98 | [0.88;1.07] | 0.622 |
| **Liver co-morbidity** (ref : no) |  |  |  |  | 0.676 |  |  |  |  |
| Metabolic** |  | 0.7 | [0.26;1.64] | 0.433 |  |  |  |  |  |
| Viral |  | 1.55 | [0.56;3.89] | 0.366 |  |  |  |  |  |
| Other |  | 2.44 | [0.11;26.43] | 0.473 |  |  |  |  |  |
| Several |  | 0.81 | [0.04;5.05] | 0.851 |  |  |  |  |  |
| **Past or present hepatocellular cancer** (ref : yes) |  | 1.02 | [0.52;2.02] | 0.965 |  |  |  |  |  |
| **MELD score** |  | 0.98 | [0.94;1.03] | 0.529 |  |  |  |  |  |
| **Liver transplant indication** (ref : decompensated cirrhosis)  Hepatocellular carcinoma |  | 1.22 | [0.62;2.41] | 0.571 |  |  |  |  |  |
| **Time on waiting period (months)** |  | 1.05 | [1.03;1.08] | **<0.001** |  |  | 1.06 | [1.03;1.1] | **<0.001** |

* Craftsmen, shopkeeper, company director

** High blood pressure and/or diabetes and/or obesity

LT: liver transplantation; MELD: Model End-Stage Liver Disease; OR: Odds Ratio; 95% CI : 95% confidence interval; ORa: Adjusted Odds Ratio**.** Ref: reference.

**Supplementary Figure 1. Patients trajectories (patients with documented alcohol consumption on list excluding patients with alcohol consumption only documented by positive testing the day of a call for LT) (N=31)**

LT n=2

AR n=1

Return to abstinence n=4

No LT n=2

No LT n=2

No LT n=15

DAC during the waiting time period (excluding LT call) n=31

TCI n=21

AR n=3

AR n=1

DAC: Documented of alcohol consumption; LT: liver transplantation; TCI tempory contraindication, AR alcohol relapse

No LT n=4

No abstinence or relapse n=7

LT n=3

Return to abstinence n=3

LT n=1

AR n=0

No abstinence or relapse n=17

LT n=2

| Discovery method |  |
| --- | --- |
| Biological n= 11 |  |
| Interview n= 18  Fortuitus n=3 |  |
| Pattern of alcohol relapse |  |
| Occasional n= 11 |  |
| Daily n= 12 |  |
| Missing data n= 6 | |

No TCI n=10

**Supplementary Figure 2. Patients trajectories (patients with alcohol consumption only documented by positive testing the day of a call for LT) (N=10)**

AR n=3

LT n=5

Return to abstinence n=6

DAC: Documented of alcohol consumption; LT: liver transplantation; TCI tempory contraindication, AR alcohol relapse

LT n=3

TCI n=7

DAC discovered the day of a LT call n=10

No abstinence or relapse n=1

AR n=0

No LT n=1

LT n=0

No LT n=1
